# Supplementary material for: Assessing the Benefits and Harms Associated with Early Diagnosis from the Perspective of Parents with Multiple Children Diagnosed with Duchenne Muscular Dystrophy
Source: Int J Neonatal Screen. 2024 Apr 15;10(2):32. doi: 10.3390/ijns10020032 (PMC11036293; doi:10.3390/ijns10020032)
Supplement: Supplementary file 1 [file IJNS-10-00032-s001.zip › Duchenne Sibling Diagnosis Survey.pdf]

Benefits and harms of early diagnosis for Duchenne: experiences of parents with multiple children with Duchenne muscular dystrophy

The purpose of this survey is to better understand parents' perspective of age at diagnosis and potential benefits and harms of a diagnosis at an earlier age. This survey focuses on parents and caregivers who have two or more children with Duchenne muscular dystrophy. Many times, in a family with two or more children with Duchenne, each child was diagnosed at a different age and may have different experiences because of the different ages at the time of diagnosis. The hope is that the information learned through this survey will help understand potential benefits and potential harms of early diagnosis through programs like newborn screening.

We anticipate that this survey will take you approximately 20 minutes to complete. Individuals are eligible to participate in this survey if they are i) 18 years of age and older, and ii) a parent, guardian, or grandparent of at least 2 children who are siblings with Duchenne muscular dystrophy. Eligible participants will receive a $20 Amazon gift card for participating in this study To receive the Amazon gift card, we will ask you to enter your email address at the end of the survey.

Qualtrics

Qualifying questions

Are you 18 years of age or older?

Are you a parent or guardian of children with Duchenne?

If yes, skip to next question

Are you a parent or guardian to 2 or more children who have Duchenne muscular dystrophy?

Demographics

1. What is your relationship to the children with Duchenne?
   - Parent
   - Guardian
   - Grandparent
2. What is your race and ethnicity
   - Drop down of race/ethnicity
3. How many children with Duchenne do you parent?
   - Drop down of numbers (skip logic will bring up a set of questions for each child. If the caregiver reports one child with Duchenne, the participant will see the not eligible screen. If the caregiver reports 2 children with Duchenne, the family will see two sets of questions 4-9. If the caregiver reports 3 children with Duchenne, the family will see 3 sets of questions 4-9, etc)

The following set of questions will ask you about your **oldest child with Duchenne**.

4. What is the current age of your oldest child with Duchenne?
   - Drop down of numbers
     - If >10, is your oldest child with DMD able to walk?
       - Yes
       - No
         - At what age did he start to use a wheelchair full-time?
5. How old was your oldest child with Duchenne when he was diagnosed? If he was diagnosed before birth or before his first birthday, please choose zero.
   - Drop down of numbers
6. For your oldest child with Duchenne, is he currently on any approved medical therapies for Duchenne or has he ever been on any approved medical therapies for Duchenne (clinical trials will be asked about in a separate question)? Medical therapies include steroids, heart medications, and exon skipping therapies.
   a. Yes
      i. Branch to Which therapies (choose as many as applicable)
         1. Corticosteroids (prednisone, Emflaza, deflazacort)
         2. Exon skipping therapies
         3. Received gene therapy (ELEVIDYS)
            a. At what age did your oldest child with Duchenne first start ANY medical therapy for Duchenne (e.g. corticosteroids or exon skipping therapies)? If he started therapies before his first birthday, please choose zero.
               i. Number drop down
   b. No
   c. I don't know
7. For your oldest child with Duchenne, is he currently in a clinical trial for Duchenne or has he ever been in a clinical trial for Duchenne? Clinical trials are research studies that test a potential medicine that usually has not been approved by the FDA.
   a. Yes

        i. At what age did your oldest child with DMD first start ANY clinical trial? If he started a clinical trial before his first birthday, please choose zero.
            1. Number drop down

8. Does your oldest child currently receive, or received in the past, any Early Intervention Services? Early Intervention Services focus on physical, learning, communication, and emotional skills and may include physical therapy, occupational therapy, or speech therapy in children 0-3 years.
    a. Yes
        i. At what age did he first start Early Intervention services?
            1. Drop down of numbers
    b. No
    c. I don’t know
9. Does your oldest child currently have or have in the past an Individualized Educational Plan (IEP) at school?
    a. Yes
        i. At what age did he first have an IEP?
            1. Drop down of numbers
    b. No
    c. I don’t know

The following set of questions will ask you about your **youngest child with Duchenne**.

10. What is the current age of your youngest child with Duchenne?
    - Drop down of numbers
        - If >10, is your youngest child with DMD able to walk?
            - Yes
            - No
                - At what age did he start to use a wheelchair full-time?
                    - Drop down of numbers
11. How old was your youngest child with Duchenne when he was diagnosed? If he was diagnosed before birth or before his first birthday, please choose zero.
    - Drop down of numbers
12. For your youngest child with Duchenne, is he currently on any medical therapies for Duchenne, or has he ever been on any medical therapies for Duchenne?
    a. Yes
        i. Branch to Which therapies (choose as many as applicable)
            1. Corticosteroids (prednisone, Emflaza, deflazacort)
            2. Exon skipping therapies
            3. In a clinical trial
            4. Received gene therapy
        ii. At what age did your youngest child with DMD first start ANY medical therapy for Duchenne (ie corticosteroids or exon skipping therapies)? If he started therapies before his first birthday, please choose zero.
            1. Number drop down
    b. No

13. For your youngest child with Duchenne, is he currently in a clinical trial for Duchenne or has he ever been in a clinical trial for Duchenne? Clinical trials are research studies that test a potential medicine that usually has not been approved by the FDA.
    a. Yes
        i. At what age did your oldest child with DMD first start ANY clinical trial? If he started a clinical trial before his first birthday, please choose zero.
            1. Number drop down
    b. No
    c. I don't know
14. Does your youngest child currently receive, or received in the past, any Early Intervention Services? Early Intervention Services focus on physical, learning, communication, and emotional skills and may include physical therapy, occupational therapy, or speech therapy in children 0-3 years.
    a. Yes
        i. At what age did he first start Early Intervention services? If he started therapies before his first birthday, please choose zero.
            1. Drop down of numbers
    b. No
    c. I don't know
15. Does your youngest child currently have or have in the past an Individualized Educational Plan (IEP) at school?
    a. Yes
        i. What age did he first have an IEP?
            1. Drop down of numbers
    b. No
    c. I don't know

16. Often in families with more than one child with Duchenne, the younger child will be diagnosed at an earlier age than the older child was initially diagnosed (e.g., big brother diagnosed at 5, little brother diagnosed at 2).

What benefits and harms did you and your family experience as a result of your younger child receiving an earlier diagnosis?

Items

	BENEFITS of early diagnosis	HARMS of early diagnosis	Neither a BENEFIT nor a HARM	Both a BENEFIT AND a HARM	Did not experience
No diagnostic odyssey (long time period between when symptom is noticed and when diagnosis is made)					

Started treatment such as corticosteroids earlier					
Prepared for what to expect					
Options for clinical trials					
Eligible for/potentially eligible for ELEVIDYS					
Access early intervention services, such as physical therapy, occupational therapy, and speech therapy					
More time to consider treatment and clinical trial options					
More time to worry					
No or shorter time period when we didn't know about the diagnosis					
Had different expectations at an earlier age because of his diagnosis					
Able to prepare for school prior to school start					
Altered parenting style					
Altered ability to bond					
No immediate plan for care					
Access medical assistance through the state or Medicaid					
Experience with older child affected experience with younger child					
OTHER (open text field)					

Open Ended questions (The questions the respondent sees will vary depending upon the responses in the above table)

17. What, if any, effects can you see from starting treatment earlier? (open text)
18. What, if any, harms can you see from starting treatment earlier? (open text)
19. Please elaborate on how knowing the diagnosis earlier was helpful for expectations and parenting. (open text)
20. Please elaborate on how knowing the diagnosis earlier was helpful with school preparations. (open text)

21. Please elaborate on how knowing the diagnosis earlier was harmful for parenting. (open text)
22. Please elaborate on how knowing the diagnosis earlier was harmful for bonding. (open text)
23. Please elaborate on how early interventions services were beneficial for your youngest child (open text)

Closing

Would you be willing to speak with a researcher in an interview about siblings with DMD?

- Yes
    - Provide email address
- No

Thank you for completing this survey. We appreciate your participation in this research. To receive the $20 Amazon electronic gift card, please provide the email address where you would like to receive the gift card.
